# Perspective: A Legal and Nutritional Perspective on the Introduction of Quinoa-Based Infant and Follow-on Formula in the EU

**DOI:** 10.1093/advances/nmab041

**Published:** 2021-04-15

**Authors:** Naomi Vita Venlet, Kasper Arthur Hettinga, Hanna Schebesta, Nadia Bernaz

**Affiliations:** Law Group, Wageningen University and Research, Wageningen, The Netherlands; Food Quality and Design Group, Wageningen University and Research, Wageningen, The Netherlands; Law Group, Wageningen University and Research, Wageningen, The Netherlands; Law Group, Wageningen University and Research, Wageningen, The Netherlands

**Keywords:** EU food law, follow-on formula, infant formula, isolated quinoa proteins, quinoa

## Abstract

Infants are vulnerable consumers and highly depend on dietary proteins for growth and development during their first months of life. Infant formula (IF) and follow-on formula (FOF) have been developed to meet these requirements, although few protein sources are currently allowed to be used. At the same time, allergies to these available protein sources are becoming more frequent. There is thus a need to explore alternative protein sources for infant nutrition. One alternative could be quinoa, which is a pseudocereal that is naturally free from gluten and has a high protein content and quality. This review assessed the composition, nutritional properties, and applicability of quinoa proteins for IF and FOF as well as the legal framework for their use in the European Union (EU). The protein quality of isolated quinoa proteins (IQPs) is relatively high compared with other plant-based proteins like rice. Besides, during the protein isolation process, unfavorable compounds are mostly removed, ensuring that the final product can comply with the maximum residue concentrations allowed. Overall, IF and FOF are strictly regulated under the Foods for Specific Groups (FSG) Regulation (EU) No 609/2013 and more research is needed before the introduction of IQP in such products is considered, but this review shows it has several promising features that warrant further investigation.

## Teaser

Statement of Significance: Quinoa is increasingly recognized as an excellent gluten-free protein source for a wide range of consumers, including infants, and isolated quinoa proteins are therefore a promising source of proteins for IF and FOF. This perspective provides new insights, using a multidisciplinary approach, on the composition, nutritional properties, and applicability of quinoa proteins, as well as the legal framework of IF and FOF in the EU.

## Introduction

Dietary proteins play an essential role as structural and functional components to maintain growth and other physiological functions in humans. Especially for infants, dietary proteins from breast milk or other protein sources, are a crucial component of the diet since they contribute to their healthy development (1–3). The recommended age of exclusive breastfeeding is variable. The WHO and UNICEF recommend exclusive breastfeeding for 6 mo, whereas the European Society for Paediatric Gastroenterology, Hepatology, and Nutrition (ESPGHAN) recommend exclusive breastfeeding for 4 mo (4, 5). For young children from the age of 6 mo, the WHO recommends nutritionally adequate and safe complementary feeding with continued breastfeeding up to the age of 2 y or more (4). Although it is important to support breastfeeding due to its health benefits to infants and young children, there are circumstances where breast milk is not available, the mother is unable to breastfeed, or breastfeeding is not appropriate. This is the case, for example, when mothers are taking medication that is contraindicated for breastfeeding, or they are HIV positive and replacement feeding is acceptable, feasible, affordable, sustainable, and safe for them and their infants (6). In these cases, infant formula (IF) and follow-on formula (FOF) can be used as a suitable breast-milk substitute (6). These formulae are specialized infant nutrition with a highly balanced composition aimed at mimicking breast milk, the golden standard, as closely as possible (7). To date, protein sources used for IF and FOF include cow milk proteins, goat milk proteins, isolated soy protein (ISP), and rice protein hydrolysates (alone or in a mixture with cow or goat milk) (8, 9).

Cow milk allergy is the most common food allergy in early life, with an estimated prevalence in developed countries ranging from 0.5% to 3% at age 1 y (10). Therefore, infants suffering from cow milk allergy, lactose intolerance, galactosemia, and/or whose parents have ethical or religious reasons for not consuming cow milk, need a different source of protein in their IF and FOF. At the time of writing, only a few other (plant-based) formulae, such as soy- and rice-based IF and FOF, are commercially available for infants who suffer from such allergies (1, 11, 12). Additionally, 10% to 14% of infants allergic to cow milk protein also showed soy protein allergy (13). Therefore, extensively hydrolyzed protein formula should be considered for infants with cow milk protein allergy (12, 13). Rice is known to be a relatively low allergenic food and there are very few reports on immediate hypersensitivity reactions upon ingestion of rice (12, 14, 15). Still, allergies to soy proteins are becoming more frequent and the nutritional adequacy of rice proteins is insufficient, which is why they are usually supplemented with essential amino acids. Therefore, it is evident that there is a need to explore the possibilities offered by alternative protein-rich crops for infant nutrition.

Chenopodium quinoa Wild has a strong potential for being such an alternative protein-rich crop, owing to both its nutritional benefits and its agricultural versatility (16). Quinoa is a pseudocereal that has been listed by the FAO as one of humanity´s most promising crops, not only for its health aspects and many uses but also as an alternative to solve problems regarding human nutrition, like inadequate protein intake (16). This plant-based source of protein may offer an alternative to animal-based proteins to a wide range of consumers, including infants, because of its high and hypoallergenic protein content and excellent essential amino acid balance (1, 16–19).

Considering quinoa's high nutritional value and potential suitability for IF and FOF, this article provides a legal and nutritional perspective on the introduction of quinoa proteins in IF and FOF. Herein, it covers the nutritional quality of quinoa proteins, the EU regulatory framework for IF and FOF, and a review of the unfavorable compounds such as antinutritional factors, contaminants, pesticide residues, and strategies to minimize these compounds in quinoa.

## Nutritional Characterization of Quinoa Protein

Quinoa is especially interesting because of its high protein content. The protein content of quinoa (12–16%) is superior to those of cereal grains and legumes, such as barley (6–13%), oat (11–15%), rice (7–9%), and maize (8–11%) (20–24). In addition, quinoa is a good source of hypoallergenic proteins and is naturally free from gluten, making it a suitable ingredient for IF and FOF (1, 19). Isolated quinoa protein (IQP) may thus serve as a suitable ingredient for infant food formulations, adding a potential novel additional protein source to the limited protein sources that may be used for IF and FOF (19, 25). Yet, it should be acknowledged that since quinoa is not a standard part of the European diet, larger amounts and frequent consumption of quinoa might enhance the development of allergies. The potential allergenicity of quinoa was studied and it was found to contain compounds capable of eliciting a hypersensitive reaction less than that of egg white and cow milk and about equal to that of soy (26).

The IQP can be isolated by several processing steps and the isolates can contain ≤90% of protein (3, 27). Besides, quinoa protein has an excellent amino acid profile and is particularly rich in lysine, histidine, and methionine, which are generally the limiting amino acids in other IF and FOF protein sources, like soy and rice (27, 28). In fact, the FAO has acknowledged the potential use of quinoa in IF and FOF because of its excellent protein composition and amino acid balance (29). There are different methods to evaluate protein quality. Two frequently used methods are the Protein Digestibility-Corrected Amino Score (PDCAAS) and the Digestible Indispensable Amino Acid Score (DIAAS). For a long time, the PDCAAS method was the suggested index by the FAO/WHO (1991) to evaluate the nutritional quality of proteins and to estimate the protein value of food for human consumption (3). However, this method has several limitations, which can lead to both under- or overestimating the value of proteins. For example, it does not take into account the antinutritional factors of plant proteins. Currently, the FAO recommends the DIAAS method for assessing protein quality (30). The DIAAS method can be considered to be a more accurate method, using the ileal amino acid digestibility (30). However, there is insufficient data on quinoa and IQP obtained with this method in practice and, therefore, this study uses the PDCAAS method to evaluate the protein quality of IQP.

**TABLE 1:** Calculated amino acid score and PDCAAS of IQP for infants (0–6 mo) and preschool children (1–2 y) compared with WHO/FAO/UNU (2007) scoring pattern for infants (0–6 mo) and preschool children (1–2 y)^1^

EAA	EAA IQP (g/100 g protein) (31)	Scoring pattern infants 0–6 mo (g/100g protein) (2)	Amino acid score for infants 0–6 mo of IQP^2^	Scoring pattern children 1–2y (g/100g protein)(2)	Calculated amino acid score for children 1–2y of IQP^2^
Histidine	3.2	2.0	1.60	1.8	1.78
Isoleucine	3.2	3.2	1.00	3.1	1.03
Leucine	6.7	6.6	1.02	6.3	1.06
Lysine	6.0	5.7	1.05	5.2	1.15
Methionine (+ cysteine)	3.5	2.8	1.25	2.6	1.35
Phenylalanine (+tyrosine)	9.4	5.2	1.81	4.6	2.04
Threonine	4.1	3.1	1.32	2.7	1.52
Tryptophan	0.95	0.85	1.12	0.74	1.28
Valine	4.2	4.3	0.98^3^	4.2	1.00^4^
PDCAAS^5^			78.67^6^		80.54^7^

1 Scoring pattern derived from the essential amino acid requirements of infants (aged 0–6 mo) and preschool-age children (1–2 y), as reported by the WHO/FAO/UNU (2007) (2). This scoring pattern is based on the amino acid pattern of human milk and is also recommended by the EFSA NDA Panel (32). EAA, essential amino acids; EFSA NDA Panel, European Food Safety Authority Panel on Nutrition, Novel Foods and Food Allergens; IQP, isolated quinoa protein; PDCAAS, Protein Digestibility Corrected Amino Acid Score; UNU, United Nations University.

2 Ratio of EAA of IQP and scoring pattern.

3 Valine is the first limiting protein in IQP according to EAA scores for infants; amino acid score (4.2/4.3=) 0.98.

4 Valine is the first limiting protein in IQP according to EAA scores for preschool children; amino acid score (4.2/4.2=) 1.00.

5 TD value of quinoa is 80.54% (33).

6 PDCAAS (0.98 × 80.54=) 78.67.

7 PDCAAS (1.00 × 80.54=) 80

In order to assess its suitability for IF and FOF, the protein quality of IQP is evaluated according to the amino acid requirements of infants and preschool children. The protein quality of IQP, according to the PDCAAS method, depends on its essential amino acid composition and digestibility (34). PDCAAS uses the amino acid score and true fecal digestibility (TD) and is calculated using the following formula:

(1) $$\begin{eqnarray*}
&&Formula\ 1:\ PDCAAS\ \left( \% \right)\nonumber\\
&&\,\,\,\,= \ \frac{{\left( {mg\ of\ first\ limiting\ amino\ acid\ in\ 100\ g\ quinoa} \right)}}{{\left( {mg\ of\ the\ same\ amino\ acid\ in\ 100\ g\ reference\ protein\ or\ scoring\ pattern} \right)}}\nonumber\\
&&\,\,\,\,\quad *\,\,TD
\end{eqnarray*}$$

The equation can be simplified as:

(2) $$\begin{equation*}
Formula{\rm{\,\, }}2:{\rm{ }}PDCAAS{\rm{ }}\left( \% \right){\rm{ }} = {\rm{ }}amino{\rm{ }}acid{\rm{ }}score{\rm{ }} \times {\rm{ }}TD
\end{equation*}$$

**Table 1** shows the calculated amino acid score and PDCAAS of IQP for infants (0–6 mo) and preschool children (1–2 y), compared with the WHO/FAO/United Nations University (UNU) (2) scoring pattern for these 2 groups. To calculate the PDCAAS, an in vitro measured TD value of 80.54 was used (33). Overall, quinoa has a high digestibility leading to a high bioavailability of the quinoa protein (20, 35). Other studies have found that the in vitro digestibility of IQP ranges from 75.3 to 95% (33, 36, 37). In vivo measured TD values for quinoa seeds were ≤92% (38). Digestibility can be affected by the extraction method of the IQP. In fact, isolation procedures have a profound influence on the structural and functional properties of the proteins, making them more susceptible and accessible for digestive enzymes and, therefore, increasing their digestibility (3).

The PDCAAS of other IF and FOF protein sources, such as casein, whey, ISP, and rice bran proteins (RBPs), were calculated and compared with IQP. Quinoa does not reach the same protein quality as casein, whey, and ISP based on the PDCAAS method (**Table 2**). Still, quinoa has a higher PDCAAS compared to rice for infants and preschool children, suggesting its suitability for use in IF and FOF for infants suffering from allergies or intolerances to cow milk proteins or ISP. Since quinoa is somewhat limiting in valine, and rice protein has a surplus of this essential amino acid, it is interesting to further investigate the possibilities of using a mixture of these plant proteins in IF and FOF.

**TABLE 2:** Calculated PDCAAS for infants (0–6 mo) and pre-school children (1–2 y) of different protein sources^1^

Protein source (reference)	PDCAAS infants (0–6mo)	PDCAAS pre-schooled children (1–2y)
Casein (28)	100	107
Whey (28)	91	102
ISP (28)	84	90
RBP (28)	76	83
IQP (33, 31)	79	81

1 Based on scoring patterns of the WHO/FAO/UNU for infants (aged 0–6 mo) and of preschool-age children (1–2 y) (2). IQP, isolated quinoa protein; ISP, isolated soy protein; PDCAAS, Protein Digestibility Corrected Amino Acid Score; RBP, rice bran protein; UNU, United Nations University.

At present, only 1 study has looked at the health effects of quinoa in infants and children and showed increased insulin-like growth factor 1 (IGF-1) concentrations in infants supplemented with quinoa-based baby food (BF), whereas the concentrations of the control group remained unchanged. Low concentrations of IGF-1 are a marker for malnutrition and increased concentrations can promote growth, body weight gain, and bone length. These positive effects were attributed to the complete essential amino acid profile of quinoa-based BF as well as its high digestibility (95.3%) (39). Even though BF derived from quinoa provides sufficient protein and other essential nutrients crucial for reducing child malnutrition, few studies have investigated the short- or long-term health consequences of the consumption of IF and FOF containing quinoa (39).

## Regulatory Framework for Infant- and Follow-on Formula

Infants, defined as children under the age of 12 mo, are considered a specific group of vulnerable consumers. Different types of nutrition exist which are specific for the developmental phase of the infant and for these products different regulations apply (40, 41). The EU legal framework distinguishes categories of food, among them the 2 groups: (1) IF and FOF, that are for infants only; and (2) processed cereal-based foods (PCBF) and BF, that are for infants and young children between the age of 1 and 3 years. The major difference between these 2 groups is that IF and FOF are the sole or a partial source of nourishment for breastfed infants and are, therefore, vital for the management of certain conditions and/or are essential to satisfy the nutritional requirements of infants (40). This research will focus on IF and FOF, however, when data is lacking for the first group references will be made to the second group.

The EU has set up the legal framework on foods for specific groups (FSG), to ensure appropriate nutritional consumption and safety of foods specifically manufactured for infants (40). IF and FOF are strictly regulated under the FSG Regulation (EU) No 609/2013 and accompanying legislative documents. These legislative documents include Directive 2006/141 and Commission Delegated Regulation (EU) 2016/127, which incorporate detailed compositional and labeling requirements (8, 9, 40). Directive 2006/141 lays down compositional and information requirements of IF and FOF, including the list of vitamins, minerals, and other substances (8). Commission Delegated Regulation (EU) 2016/127, supplementing the FSG Regulation, replaced these requirements in February 2020 (9). IF and FOF may only be placed on the market if they comply with Regulation (EU) 2016/127. This regulation only allows the following compositional requirements: IF and FOF based on cow milk proteins, goat milk proteins, ISP, and protein hydrolysates (alone or in a mixture with cow or goat milk). Hence, to date, quinoa protein cannot be used in IF and/or FOF (9).

The FSG Regulation stipulates that the foods may contain substances that are considered as novel food or a novel food ingredient under the applicable EU legislation, as long as they fulfill the conditions for being placed on the market under Regulation (EC) No 258/97, which was replaced in 2015 by the new Novel Food Regulation (EU) 2015/2283 (40, 42). Substances intended for IF and FOF must be assessed in accordance with the rules of the Novel Food Regulation when they fall within the definition of novel food set out therein (42). A novel food is a food that has not been consumed to a significant degree by humans in the EU before 15 May, 1997. Novel foods can be newly developed and innovative food; food produced using new technologies and production processes; as well as food, which is or has been traditionally eaten outside of the EU (42). Quinoa grains or fruits (nuts) as a food ingredient is known in the EU and has been consumed to a significant degree before 15 May, 1997 and is therefore, not classified as a novel food (43). However, under Article 3 of Regulation (EU) 2015/2283, foods that for instance result from a production process not used for food production within the Union before 15 May, 1997 are also considered novel food (42). Presumably, quinoa would be included to IF and FOF as protein isolate, and therefore its processing and end product will render it a novel food. This results in 2 legal burdens, namely (1) the inclusion in the FSG Regulation and (2) authorization as a novel food.

The procedure for authorizing the placement on the market of a novel food can start either on the Commission's initiative or following an application to the Commission by an applicant (e.g. food business operators) (42). The suitability of an ingredient can be demonstrated through a systematic review of data relating to the expected benefits and to safety considerations as well as, where necessary, appropriate studies, performed following generally accepted expert guidance on the design and conduct of such studies (8, 9). For example, a study conducted by Nestlé showed that the protein content in FOF based on cow milk should be lowered. This was a prospective, open-label, multicenter, single-arm, 12-mo study (44). Accordingly, the European Food Safety Authority (EFSA) has delivered a scientific opinion on the safety and suitability for the use of FOF with a protein content of ≥1.6 g/100 kcal (45). This example shows the amplitude of a study conducted to lower the protein content of an already frequently used protein source in FOF, namely cow milk. Thus, when introducing a new IF and/or FOF based on quinoa on the EU market, business operators will have a responsibility to demonstrate the suitability of IF and FOF to the competent national authorities. Yet, these conditions can be very demanding and require an economic risk of, e.g. the protein supplier and the infant nutrition manufacturer. As this process requires time and resources, which is a risk (smaller) manufacturers may not be willing to take, the development of quinoa-based IF and/or FOF could be set back. More research regarding the nutritional adequacy as well as potential health benefits and disadvantages of quinoa in IF and FOF could guide business operators in taking the next steps.

## Antinutritional Factors in Quinoa

Antinutritional factors are a class of compounds present in plant foods and could reduce their nutritional value by interfering with digestibility, absorption, and utilization of nutrients. The most common examples are trypsin inhibitors, phytic acid, nitrates, and saponins (46). Trypsin inhibitors might interfere with the digestion of proteins in the intestinal tract (1). For this reason, the EFSA Panel on Nutrition, Novel Foods and Food Allergens (NDA Panel) considers that concentrations of trypsin inhibitors in IF and FOF from ISP should be kept as low as feasible (1). The concentration of trypsin inhibitors in quinoa seeds ranged from 1.36 to 5.04 units trypsin inhibitor (UTI)/mg, which is much lower than in soybean (24.5 to 41.5 UTI/mg) (47, 48). This could suggest a higher digestibility of quinoa compared with soybean. However, trypsin inhibitors often have a limited effect in human nutrition because they are thermolabile and are usually destroyed under normal conditions of domestic or industrial food preparation (46). A study by Khattab and Arntfield (49) showed that this is also the case for trypsin inhibitors in all pulses and legumes, demonstrating that the inactivation of this antinutritional factor in quinoa can be obtained by general food preparation techniques.

In the outer layer and in the endosperm of plant proteins, another common antinutritional factor may be present, namely phytate. Phytates are known for compromising the bioavailability of minerals such as calcium, iron, magnesium, zinc, as well as starch, protein, and enzymes (48). Currently, the only permitted source of plant protein in IF and FOF, which is ISP, contains ∼1–2% phytate. ESPGHAN recommends that soy phytates in IF should be effectively reduced by phytase treatment or via precipitation methods (50). Research showed that reducing the phytic acid content in IF and FOF by half or completely, improves zinc and iron absorption (50). The content of phytic acid can also be reduced by washing, germination, and further processing. However, since it is also located in the endosperm, the content of phytic acid is only lowered by 30% with these treatments (48). Other animal studies have shown that quinoa consumption has no adverse effect on the incorporation of calcium in the bones, nor on iron absorption (26, 48).

Nitrates are another category of antinutritional factors that are present in all plants and are essential for their growth, which can also originate from fertilizers in the soil. In the human body, nitrates interfere with vitamin A metabolism and in the functions of the thyroid gland. In addition, they may be transformed into carcinogenic compounds (46). Besides, infants aged under 3 mo are thought to be more vulnerable than adults to this particular toxic effect of nitrate (49). A study by Lopes et al. (51) found nitrate values in whole quinoa flour of 632.6 mg/kg. Regulation (EU) 1258/2011 sets no maximum concentrations for nitrates in IF and FOF but it states that the allowed nitrate content in PCBF and BF is 200 mg/kg (52). This indicates that nitrate concentrations in quinoa are 3 times higher, which could restrict the use of quinoa in PCBF and BF. This is even more problematic for IF and FOF, because PCBF and BF contain a mixture of different ingredients, whereas IF and FOF would mainly consist of quinoa proteins. Yet, nitrate concentrations in the soil can vary by 3 to 4 orders of magnitude and specific farming techniques could have a lowering effect on the nitrate content (53–55). Dutch organic low-saponin quinoa seeds show more promising nitrate results (351.0 mg/kg), yet still higher than the allowed values set out in Regulation (EU) 1258/2011 (GreenFood50 B.V.). Little is known about the reduction of nitrate in quinoa plants, however, some approaches may be adopted that are known to reduce nitrate concentrations in vegetables: a balanced fertilization program for the crop; replacing nitrate-based fertilizers with ammonium-based nitrogenous fertilizers; selection among the available genotypes/cultivars; and breeding of new cultivars that do not accumulate nitrate (55). In addition, it has been shown that cooking legumes, such as peas, in water can reduce nitrate by ≥70%, but whether this can be applied to quinoa seeds has not been studied yet (56).

The quinoa grain also contains a bitter coating called saponin, which is generally present in the outer layers of the grain and protects it from birds, and fungal and bacterial attacks (20, 57). Saponins have traditionally been considered antinutritional factors, decreasing mineral and vitamin bioavailability and absorption (58–60). The content of saponins in the low-saponin variety of quinoa was found to be between 0.02 and 0.04% (on dry weight) (61). These values are below those found in soybeans (2%) (62). Researchers have investigated the ability of infants to digest and absorb soy saponins, however, their effects are largely unknown and more long-term studies are needed to evaluate the safety of saponin intake in infants (63). To date, there are no reports in the literature that show harmful effects of feeding infants with soy-based formula, which could give an indication on the safety of the saponin concentration in quinoa, as they contain lower concentrations than soy (63). In any case, saponins can be removed through specific processing methods (46). Depending on the quinoa variety, washing is one effective method to reduce the saponin content, (64). However, the new low-saponin quinoa varieties, cultivated in Europe, might provide developments in this area, since they are low in saponins, for which no extra processing steps are needed, and have an increased digestibility (35).

Overall, these antinutritional factors are important to consider when using quinoa as the main protein source and should be removed as much as possible to enhance the protein quality (3). To achieve this, many processing methods can significantly reduce the concentration of antinutritional factors. Besides, IQP will most likely be used in IF and FOF and not the unrefined quinoa flour. This step of producing protein isolates from quinoa may reduce or completely remove these unfavorable compounds (65). For example, protein isolates of legumes, like cowpea, showed no traces of phytic acid and a significantly lower amount of trypsin inhibitors compared with the raw seeds and flours (3, 66). More research on the antinutritional factors of IQP, and methods to further reduce them, would provide essential insights in its use for IF and FOF.

## Quinoa and EU Maximum Allowed Pesticide and Contaminant Concentrations in Infant and Follow-on Formula

Due to the possible contamination of the raw material or from the production chain, IF and FOF can contain harmful substances such as pesticide residues and/or contaminants. Regulation 1881/2006 sets out the maximum concentrations of heavy metals and mycotoxins for IF and FOF in the EU (67). A study by Vollmannová et al. (68) showed that quinoa seeds are prone to take up high concentrations of cadmium and lead. Concentrations of lead in organically cultivated Dutch low-saponin quinoa seeds (0.022 mg/kg) were below maximum concentrations for IF and FOF (powder: 0.05 mg/kg; liquid: 0.1 mg/kg) (**Supplemental Table 1**). However, cadmium concentrations in organic quinoa seeds (0.022 mg/kg) were slightly higher than the allowed maximum levels (powder 0.01 and liquid 0.005 mg/kg for milk based; powder 0.020 and liquid 0.010 mg/kg for soy based). It must be emphasized that these maximum concentrations are established for milk- and soy-based products and not for quinoa. It is necessary to permanently monitor the soil, as well as the plant content for heavy metals and apply measures in order to reduce contamination in quinoa seeds (68). Besides, it is important to note that IQP is not the sole ingredient in formulae, therefore, research should be conducted on how much residue ends up in the final product.

Maximum mycotoxin concentrations in IF and FOF are not determined, except for aflatoxin M1 and ochratoxin (67). Aflatoxin M1, also known as the “milk toxin,” is formed through the ingestion of aflatoxin B1 by mammals and ends up in the milk (69). Therefore, this mycotoxin is not applicable for quinoa. Aflatoxin B1 can easily occur on feeds and foods during growth, harvest, and storage (69). Dutch organic low-saponin quinoa seeds (≤0.01 μg/kg) do not exceed the allowed maximum concentration for aflatoxin B1 in PCBF (0.10 μg/kg), as well as ochratoxin (≤0.1 μg/kg) in IF and FOF (0.5 μg/kg) (Supplemental Table 1). Accordingly, a study by Pappier et al. (70) also found no mycotoxins in quinoa seeds as natural contaminants. They hypothesized that quinoa is similar to other small pseudograins (e.g. amaranth), which are less susceptible to mycotoxin contamination than larger sized grains.

Delegated Regulation (EU) 2016/127 stipulates prohibited and maximum residues of pesticides for IF and FOF in the EU (9). A very low residue limit of 0.01 mg/kg for all pesticides is set and, in addition, more severe limitations are set for a small number of pesticides or metabolites of pesticides (9). No residues of these pesticides were detected in Dutch organically cultivated low-saponin quinoa seeds (**Supplemental Table 2**). Besides, pesticides which are prohibited in the agricultural products intended for the production of IF and FOF, were not detected in Dutch organic low-saponin quinoa seeds (**Supplemental Table 3**). Notably, these values have been observed in organically cultivated quinoa seeds and can thus deviate for nonorganic cultivations.

In general, whole (pseudo-)grains, such as quinoa, are more prone to pesticide residues and contaminants than refined cereals, since they contain all grain components, such as the endosperm, bran, and germ. The outer layer of quinoa grains is more likely to be exposed to contaminants such as heavy metals, mycotoxins, and/or pesticides (71). During the isolation process of IQP, other grain compounds are separated, thereby possibly removing most of the contaminants and pesticide residues. In addition, this review has investigated the concentration of contaminants and pesticide residues in quinoa cultivated for adult consumption and not specifically intended for infant food. Stricter cultivation and soil requirements apply for agricultural products intended for infant nutrition, ensuring its compliance with EU contaminant and pesticide concentrations.

## Conclusions and Recommendations

Infants suffering from intolerances or allergies to common protein sources in IF and FOF, would benefit from an alternative protein source in these products. This article has highlighted the excellent protein quality and suitability of IQP, specifically for IF and FOF. Still, its application in IF and FOF faces 2 legal burdens, namely the inclusion in the FSG Regulation and authorization as a novel food. Most of the essential amino acids in quinoa are sufficient according to the FAO/WHO suggested requirements for infants and children. Furthermore, this study has shown that the protein quality of IQP is comparable to that of rice, yet lower than that of milk proteins. Due to its complementary amino acid profile, a mixture of these plant proteins could be considered when developing a plant-based IF or FOF, with both proteins being low in allergenicity. Although it is likely that most unfavorable compounds, such as antinutritional factors, contaminants, and pesticides are less frequent in IQP, their concentrations should be monitored closely and be kept as low as possible. IQPs are a promising source of proteins for IF and FOF in the EU and the evaluation of their nutritional suitability for infants, in vivo digestibility, and antinutritional factor concentrations is desirable for the following stages of research.

## Supplementary Material

nmab041_Supplemental_File

## Notes

Supported by GreenFood50 B.V., Wageningen.

Author disclosures: The authors report no conflicts of interest.

Supplemental Tables 1–3 are available from the “Supplementary data” link in the online posting of the article and from the same link in the online table of contents at https://academic.oup.com/advances/.

Perspective articles allow authors to take a position on a topic of current major importance or controversy in the field of nutrition. As such, these articles could include statements based on author opinions or point of view. Opinions expressed in Perspective articles are those of the author and are not attributable to the funder(s) or the sponsor(s) or the publisher, Editor, or Editorial Board of *Advances in Nutrition*. Individuals with different positions on the topic of a Perspective are invited to submit their comments in the form of a Perspectives article or in a Letter to the Editor.

Abbreviations used: BF, baby food; DIAAS, Digestible Indispensable Amino Acid Score; EFSA, European Food Safety Authority; ESPGHAN, European Society of Paediatric Gastroenterology, Hepatology, and Nutrition; EU, European Union; FOF, follow-on formula; FSG, foods for specific groups; IF, infant formula; IGF-1, insulin-like growth factor 1; ISP, isolated soy protein; IQP, isolated quinoa protein; PDCAAS, Protein Digestibility-Corrected Amino Score; PCBF, processed cereal-based foods; RBP; rice bran protein; TD, true fecal digestibility; UNU, United Nations University; UTI, unit trypsin inhibitor.
