# Supplementary material for: Perspective: A Legal and Nutritional Perspective on the Introduction of Quinoa-Based Infant and Follow-on Formula in the EU
Source: Adv Nutr. 2021 Apr 15;12(4):1100–7. doi: 10.1093/advances/nmab041 (PMC8382516; doi:10.1093/advances/nmab041)
Supplement: nmab041_Supplemental_File [file nmab041_Supplemental_File.docx]

**Supplemental Table 1** The maximum level of heavy metals and mycotoxins according to Regulation 188/2006 compared to levels in Dutch organic low-saponin quinoa seeds^1^

| Contaminant | Unit | Measured level of contaminants in quinoa^2^ | Maximum level according to EU Reg. 1881/2006 IF and FOF (65) | Maximum level according to EU Reg. 1881/2006 PCBF (65) |
| --- | --- | --- | --- | --- |
| HEAVY METALS | | | | |
| Lead | mg/kg | 0.022 | Powder: 0.050 | 0.050 |
|  |  |  | Liquid: 0.10 |  |
| Cadmium | mg/kg | 0.022 | Liquid (soy): 0.010 | 0.040 |
|  |  |  | Powder (milk): 0.010 |  |
|  |  |  | Powder (soy): 0.020 |  |
|  |  |  | Liquid (milk): 0.005 |  |
| Arsenic | mg/kg | < 0.03 | 0.10 | 0.10 (rice intended for the production of food for infants and young children) |
| Mercury | mg/kg | < 0.01 | ND | ND |
| Chrome | mg/kg | 0.054 | ND | ND |
| Copper | mg/kg | 5.7 | ND | ND |
| Zinc | mg/kg | 39 | ND | ND |
| Nickel | mg/kg | 0.43 | ND | ND |
|  |  |  |  |  |
| MYCOTOXINS | | | | |
| Aflatoxin B1 | μg/kg | ≤ 0.01^3^ | ND | 0.10 |
| Aflatoxins B1+B2+G1+G2 | μg/kg | ≤ 4^3^ | ND | ND |
| Aflatoxin M1 | μg/kg | NA for quinoa | 0.025 | ND |
| Ochratoxin | μg/kg | ≤ 0.01^3^ | 0.5 | 0.5 |
| Zearalenon | μg/kg | < 1 | ND | 20 |
| Deoxynivalenol | μg/kg | < 10 | ND | 200 |

^1^ND, Not determined; NA, not applicable.
^2^Levels in Dutch organic low-saponin quinoa seeds obtained from GreenFood50 B.V., Wageningen.
^3^Value below detection limit of 0.003 mg/kg.

**Supplemental Table 2** Pesticides which shall not be used in agricultural production intended for the production of infant formulae and follow-on formulae according to Delegated Regulation (EU) 2016/127 compared to pesticide residues in Dutch organic low-saponin quinoa seeds^1^

| Chemical name of the substance (residue definition) (9) | Pesticide residue in quinoa (mg/kg)^2^ |
| --- | --- |
| Aldrin and dieldrin, expressed as dieldrin | Not detected |
| Disulfoton (sum of disulfoton, disulfoton sulfoxide and disulfoton sulfone expressed as disulfoton) | Not detected |
| Endrin | Not detected |
| Fensulfothion (sum of fensulfothion, its oxygen analogue and their sulfones, expressed as fensulfothion) | Not detected |
| Fentin, expressed as triphenyltin cation | Not detected |
| Haloxyfop (sum of haloxyfop, its salts and esters including conjugates, expressed as haloxyfop) | Not detected |
| Heptachlor and trans-heptachlor epoxide, expressed as heptachlor | Not detected |
| Hexachlorobenzene | Not detected |
| Nitrofen | Not detected |
| Omethoate | Not detected |
| Terbufos (sum of terbufos, its sulfoxide and sulfone, expressed as terbufos) | Not detected |

^1^Pesticides are considered not to have been used if their residues do not exceed a level of 0.003 mg/kg (9).
^2^ Levels in Dutch organic low-saponin quinoa seeds obtained from GreenFood50 B.V., Wageningen (detection limit of 0.003 mg/kg).

**Supplemental Table 3** Specific maximum residue levels of pesticides or metabolites of pesticides in infant formulae and follow-on formulae according to Delegated Regulation (EU) 2016/127 compared to pesticide residues in Dutch organic low-saponin quinoa seeds^1^

| Chemical name of the substance | Maximum residue level (mg/kg) (9) | Residue-level in quinoa (mg/kg)^1^ |
| --- | --- | --- |
| Cadusafos | 0.006 | Not detected |
| Demeton-S-methyl/demeton-S-methyl sulfone/oxydemeton-methyl (individually or combined, expressed as demeton-S-methyl) | 0.006 | Not detected |
| Ethoprophos | 0.008 | Not detected |
| Fipronil (sum of fipronil and fipronil-desulfinyl, expressed as fipronil) | 0.004 | Not detected |
| Propineb/propylenethiourea (sum of propineb and propylenethiourea) | 0.006 | Not detected |

^1^ Levels in Dutch organic low-saponin quinoa seeds obtained from GreenFood50 B.V., Wageningen (detection limit of 0.003 mg/kg).
